# Predicting Depression From Language-Based Emotion Dynamics: Longitudinal Analysis of Facebook and Twitter Status Updates

**DOI:** 10.2196/jmir.9267

**Published:** 2018-05-08

**Authors:** Elizabeth M Seabrook, Margaret L Kern, Ben D Fulcher, Nikki S Rickard

**Affiliations:** ^1^ Monash Institute of Cognitive and Clinical Neurosciences School of Psychological Sciences Monash University Melbourne Australia; ^2^ Centre for Positive Psychology The Melbourne Graduate School of Education The University of Melbourne Melbourne Australia

**Keywords:** automated text analysis, depression, Facebook, Twitter, emotions, variability, instability

## Abstract

**Background:**

Frequent expression of negative emotion words on social media has been linked to depression. However, metrics have relied on average values, not dynamic measures of emotional volatility.

**Objective:**

The aim of this study was to report on the associations between depression severity and the variability (time-unstructured) and instability (time-structured) in emotion word expression on Facebook and Twitter across status updates.

**Methods:**

Status updates and depression severity ratings of 29 Facebook users and 49 Twitter users were collected through the app *MoodPrism.* The average proportion of positive and negative emotion words used, within-person variability, and instability were computed.

**Results:**

Negative emotion word instability was a significant predictor of greater depression severity on Facebook (*r*_s_(29)=.44, *P*=.02, 95% CI 0.09-0.69), even after controlling for the average proportion of negative emotion words used (partial *r*_s_(26)=.51, *P*=.006) and within-person variability (partial *r*_s_(26)=.49, *P*=.009). A different pattern emerged on Twitter where greater negative emotion word variability indicated lower depression severity (*r*_s_(49)=−.34, *P*=.01, 95% CI −0.58 to 0.09). Differences between Facebook and Twitter users in their emotion word patterns and psychological characteristics were also explored.

**Conclusions:**

The findings suggest that negative emotion word instability may be a simple yet sensitive measure of time-structured variability, useful when screening for depression through social media, though its usefulness may depend on the social media platform.

## Introduction

### Extending How Social Media Language Predicts Depression

*“With as much as we have learned about emotions, it is as if we have been taking still photos of a dance”* [1].

Social media is used in different ways by different people, but for many individuals, status updates provide snapshots of their lived experience. Studies to date have primarily considered how the relative frequency of words indicating positive and negative emotion relate to other characteristics such as mental health status, or which words (or set of words) best predict different outcomes. Such studies indicate that the frequent expression of negative emotion words in status updates can accurately identify individuals experiencing symptoms of depression [2-6]. However, an individual’s mental health is reflected by more than just the average frequency or the type of words used; variability in emotional expression over time might also provide significant insights. In the current research, fluctuations in emotional expression over time is explored as another window of insight into the psychological health of social media users.

### Depression in Status Updates on Social Media

Depression, including major depressive disorder (MDD) and dysphoria, are characterized by persistent low mood (including sadness or emptiness) or anhedonia (inability to experience pleasure from activities that are usually enjoyable) [7]. At a broad level, the frequent expression of negative affect within social media status updates has been associated with higher levels of depression symptoms [2,3,5,8-11]. Frequently expressing positive affect, on the other hand, tends to be associated with lower levels of depression and greater levels of well-being [9,12,13]. The link between expressed emotion in status updates and mental health is unsurprising considering that expressing current emotion and venting frustration have been reported to be a primary purpose for many users posting on Facebook [14]. Indeed, negative and positive emotional language has been observed to occur in approximately 34% and 55% of status updates on Facebook, respectively [15]. Adding to this, depressed individuals have also been shown to post content more frequently than nondepressed persons [16], and changes in depression severity may be signaled by increases in posting behavior on social media [17]. Combined, the time-structured features and emotional features of status updates may provide insights into the depression status of social media users.

Several studies have sought to code the content of social media posts for depression disclosures [6]. For instance, Moreno et al [3] demonstrated that status updates on Facebook with references to depression symptoms such as hopelessness were positively correlated with self-reported depression symptoms. Others extended this by describing the linguistic characteristics of depression in posts and developing coding schemes to identify depression-indicative tweets or status updates [2,4,8,18]. Although specific topics, keywords, and linguistic features (especially negative emotions) are able to identify depression-indicative posts with high accuracy, many of these features may also be present in posts that are nonindicative of depression (low specificity). For example, Mowery et al [18] found considerable signal discrepancies—over 70% of tweets identified in their sample containing words related to depression were not actually indicative of depression. Thus, although negative emotion words correlate with the presence of depressive symptoms, it is a noisy and imprecise metric.

This highlights the need to move beyond the frequency of emotional language alone toward other online behavioral indices that may better differentiate depressed and nondepressed individuals. Due to the time-sensitive nature of social media data, examining the dynamic movement of emotion across status updates may provide an additional avenue to tap into the nuanced cognitive-emotional processes underlying depression and may provide a more specific index of maladaptive emotional functioning.

### The Emotion Dynamics of Depression

A major change in functioning associated with the onset of depression is the ability to effectively regulate emotion. Although the capacity for emotion to vary over time is adaptive and may contribute to psychological well-being, higher levels of emotion variability, especially of negative emotions, have been linked to depression [1,19]. For example, individuals who experience intense negative affect reactivity in response to daily stressors are at greater risk of developing depression [20-22]. This experience is supported by young people’s qualitative accounts of depression, where depression is reported to “[take] over during times of vulnerability such as stress or fatigue” [23]. Negative cognitive biases also contribute to emotion variability in depression. Excessive focus on personal distress (rumination) may lead to persistent experiences of severe negative affect and difficultly regulating mood away from negative states [24,25]. The combination of cognitive-emotional processes results in emergent emotion patterns that can manifest at inappropriate times and in inappropriate ways in response to internal and external events. Maladaptive patterns of emotion build over time to place the individual at an increased risk for depression onset and maintenance [19,26-28].

The emotion variability in depression described above has predominantly been operationalized in two ways. First, variability may be operationalized as within-individual variability as *iSD*, an individual’s SD of emotion expression. Like the mean, variability may best be viewed as a *trait-like* measure of emotion expression, as it provides a single number that summarizes the overall variability in affect for an individual across their recording period but ignores time-structured information [29].

A second operationalization of variability describes emotional instability and uses the mean squared successive difference (MSSD) statistic [30] that quantifies differences between consecutive observations of emotion [1]. This time-structured measure of variability uses the temporal ordering of measurements to quantify the magnitude of incremental changes in emotion [30-32]. Crucially, unlike *iSD*, where the same result would be obtained if the same set of emotion expression observations are shuffled through time, the MSSD is sensitive to the time-ordering of observations. For example, for the same distribution of negative emotion values (and thus the same *iSD*), negative emotion increasing in small incremental steps from mild to severe would result in a small MSSD value, whereas negative emotion alternating (or swinging) between mild to severe would result in a large MSSD value. In this way, MSSD captures the temporal instability of positive or negative affect.

Negative affect instability, as measured by the MSSD, has been linked to more severe depression symptoms across several studies and has been identified as a concomitant and early indicator of depression [25,31,33-36]. It has been shown to be a significant risk factor for more frequent and severe suicidal ideation [35] and may be a unique underlying emotion pattern in depression. Negative affect instability has been shown to continue to predict depression when average negative affect and the frequency of negative event exposure are held constant [25,36]. In addition, reductions in negative affect have been shown to be greater for depressed individuals in response to positive events when compared with those who are not depressed, further contributing to potential moment-to-moment variability [37].

**Table 1:** Definitions and conceptual overlap of variability, instability, and inertia.

Name	Definition	Operationalization	Conceptual overlap
Variability	The amplitude of an individual’s emotion. This is time-unstructured, referring to the “general dispersion” of scores.	Within-person SD (*iSD*)	Variance
Instability	The amplitude of moment-to-moment changes in emotion. This is time-structured, where higher scores indicate greater variance and less positively correlated between observations.	Mean squared successive difference	Variance, time-dependency
Inertia	How well a previous emotional state predicts the next emotional state. This is time-structured, where greater correlation coefficient indicates increased temporal dependency between observations.	Autocorrelation coefficient	Time-dependency

Bowen et al [33] recently aggregated 4 studies to examine the differences in mood instability between depressed and nondepressed individuals. Participants completed daily mood diary ratings of negative and positive mood upon awakening and before bedtime for 1 week. Depressed individuals experienced greater negative mood instability over the course of the week compared with nondepressed individuals. Depressed individuals also reported greater severity in negative mood than the nondepressed group, suggesting depression is characterized by both persistent low mood and more extreme daily variation in its severity.

Although depression has also been associated with a blunted emotional response to stimuli and smoother emotional experiences from day to day (inertia) [32,38,39], variability and instability span major categories of emotion dynamics as they relate to depression and are the focus of this study. Table 1 outlines the definitions of variability, instability, and inertia and describes their conceptual overlap. To best examine the unique associations that emotion dynamic patterns have with mental health, it has been recommended that the conceptual overlap between these measures be taken into account and controlled for in analyses [32], as is done in this study.

### Social Media and Emotion Dynamics

Emotion dynamics may provide important insights into the “building blocks” of depression [28], but it is also challenging and time-intensive to collect adequate longitudinal emotion data. Current approaches rely on experience sampling methods (ESMs), where an individual inputs emotion information throughout the day [1,28,40]. Although the potential burden and invasiveness of real-time data collection has been significantly reduced by incorporating new and familiar technologies into ESM design (eg, smartphones) [41,42], the need to respond to automated prompts creates a context that is different than normal daily activities. Furthermore, these methodologies may not be practical for large-scale monitoring of public mental health.

Social media may be a powerful complementary tool. Considering the frequent use of emotion language in status updates that relate to current experiences [14,15], for a large proportion of the population, social media can provide unobtrusive access to time-sensitive and ecologically valid samples of expressed emotion [2,43-45]. Diurnal and seasonal variation in depression severity have been observed at a population [2] and individual level [4] on social media. In these studies, an increase in the linguistic features predictive of depression risk was observed from day to night and from summer to winter months. Using the social media platform Reddit, De Choudhury et al [46] considered transitions from mental health subreddits only to also using a suicide support subreddit. Findings suggested that a shift from commonly expressed sentiment (ie, the average) may represent a change toward better or poorer mental health, particularly where the magnitude of the change is more pronounced. Although observations of emotion variability and instability are yet to be applied to social media as a means of automatically screening for individuals at risk of depression, it is likely that in addition to the ability to track macro-level changes in depression on social media, microlevel changes in emotion (emotion variability) relevant to mental health may also be observable.

### This Study

Evidence is mounting to suggest that emotion patterns, including variability and instability, are early indicators for depression risk [19], and there is a need to utilize scalable and unobtrusive means of collecting emotion data to effectively apply these insights to monitoring public mental health. Targeting emotion variability and instability as indicators of maladaptive emotional functioning in depression is a clear area in need of further research on social media. To date, most studies examining emotion language on social media and depression have provided a static view of emotion by compressing the variation of social media language over time into an overall average, stripping the data of what could be meaningful patterns in temporal variation of emotion expression. Although the average emotion that individuals express on platforms such as Facebook and Twitter can provide accurate and sensitive insights into the presence of depression, the variability in emotion across posts has yet to be examined as a legitimate individual difference (rather than measurement error) that may be indicative of depression severity.

Taking advantage of the time-sensitive and naturally occurring data available from status updates on Facebook and Twitter, the major aim of this study was to demonstrate the feasibility of using status update emotion variability and instability as an indicator of depression severity (measured by the Patient Health Questionnaire-9, PHQ-9) [47]. It also aimed to examine if emotion instability was related to depression when controlling for its conceptual overlap with variability.

It was hypothesized that (1) Self-reported depression severity would be positively related to negative emotion word variability and instability across status updates, (2) Self-reported depression severity would be positively related to the average proportion of negative emotion words used and negatively related to the average proportion of positive emotion words used in status updates on Facebook and Twitter, (3) Negative emotion word instability would remain positively associated with depression severity when controlling for negative emotion word variability, (4) The emotion word patterns and their association with depression would be consistent across Facebook and Twitter, and (5) Depression severity would be positively associated with the average number of status updates per day and negatively associated with the time interval between consecutive status updates (ie, shorter periods of time between posts).

## Methods

### Participants

This study used a subset of users from the *MoodPrism* project. *MoodPrism* is a mood-tracking app that collects data and provides engaging feedback to its users on their mood, mental health, and well-being [42]. *MoodPrism* is available for download on the iPhone operating system (iOS, Apple Inc) and Android stores for smartphone. All procedures were approved by the Monash University Human Research Ethics Committee.

Participants were recruited by convenience sampling, community engagement, and targeted online advertising (smartphone owner, interested in mental health, lives in Australia)*.* To be included in this study, participants had to download the *MoodPrism* smartphone app, complete the depression severity index available in the app, and opt in to contribute their Facebook or Twitter data, which were automatically collected by the *MoodPrism* app. A minimum of 10 status updates over a minimum period of 7 days was required for the inclusion of a participant, to allow robust calculation of emotion word variability and instability over time. Additionally, status updates were only included if they occurred within the 12 months before the administration of the PHQ-9. For the Twitter data, only original tweets (not retweets) were used. Although retweets may reflect values or interests of a user and include topics similar to self-authored tweets [48], they also introduce ambiguity about the author’s sentiments [49,50]. Furthermore, the Facebook data did not have a similar repost function, such that self-authored tweets provide a more direct behavioral comparison.

Of the 1518 users who downloaded the *MoodPrism* app from April 2016 to May 2017, 223 (14.70%) provided permission to access their social media data. After applying the inclusion criteria outlined above, 3 participants were found to have contributed both Facebook and Twitter data. These participants had a greater number of language samples on Facebook than on Twitter, and thus, were allocated to the Facebook group. A final sample of 29 Facebook users (11 males, 17 females, 1 missing) with a mean age of 32.77 years (SD 8.40, range=19-45, n=22) and 49 Twitter users (16 males, 32 females, 1 missing) with a mean age of 35.03 years (SD 12.33, range=16-57, n=39) was obtained. Participants were well educated, with 35% (10/29; Facebook) and 41% (20/49; Twitter) of participants having completed tertiary education. Chi-square tests revealed no significant differences gender or education between the included samples and those who had opted in to contribute social media data but did not meet the inclusion criteria. Independent samples *t* tests revealed no significant differences between groups in age. There were also no significant differences between the included Facebook (n*=* 29) and Twitter (n*=* 49) samples in age, gender, or education.

### Procedure

After downloading and opening *MoodPrism,* participants read an explanatory statement and provided their consent to participate. They then provided an additional opt-in consent to share their Facebook or Twitter data. If consent was provided, the *MoodPrism* app then automatically extracted the participant’s previous status updates on Facebook or Twitter and repeated this extraction for all new status updates posted while *MoodPrism* was installed on the participant’s smartphone. Status updates were processed locally on the participant’s smartphone through the app, pulling out the time, total word count, and number of positive and negative emotion words, and then these summaries were uploaded to a secure server every 24 hours, at which point the status update content was permanently deleted from *MoodPrism* ’s memory. Thus, the app provided summaries of how often emotion words were expressed, but the actual status updates were unavailable for analysis.

Participants additionally completed several blocks of questionnaires on *MoodPrism*. These blocks included demographic items collecting gender and age information and measures assessing mental health, personality, and other psychological characteristics (see [40] for the full list of measures). Blocks could be completed in any order at a time of the participants’ convenience and collectively took an average of 37 min 14 s (SD 11 min 33 s) to complete.

### Measures

All data for this study was collected via the *MoodPrism* app*.* Depression symptom severity was measured by the PHQ-9 [47], a 9-item self-report measure for depression that indicates the severity of symptoms experienced over the previous 2 weeks. Each item on the PHQ-9 (eg, “Feeling down, depressed, or hopeless”) is rated from 0=“Not at all” to 3=“Nearly every day.” These ratings are summed to create a total score ranging from 0 to 27, where higher scores indicate greater severity of depression symptoms. The PHQ-9 has been validated for use in the general population (Cronbach alpha=.87) [51] and in primary care settings (Cronbach alpha=.89) [47]. The internal reliability of the PHQ-9 was good for both the Facebook (Cronbach alpha=.87) and Twitter (Cronbach alpha=.90) samples.

Language samples from Facebook and Twitter were obtained by *MoodPrism* via the Facebook and Twitter application programming interfaces, as detailed in Rickard et al [40]. The period of posts sampled per participant between their first status update and the administration of the PHQ-9 ranged from 9 to 365 days (Facebook mean 170.69, SD 116.05; Twitter mean 145.61, SD 124.97).

*MoodPrism* ’s automated scripts identified the total number of *words* and positive and negative emotion words in the status updates. *Words* on social media include both normal words and variants (eg, misspellings, emoticons, and abbreviations) that are common on social media [43]. The scripts incorporated the positive and negative emotion dictionaries of the *Linguistic Inquiry and Word Count 2007* (*LIWC 2007*) [52], a widely used corpus of dictionaries commonly used for language analysis. The *LIWC 2007* dictionaries were supplemented by common emoji’s and internet slang that indicated positive or negative emotion (see Multimedia Appendix 1). Although not definitive, these inclusions were made to better reflect the language used on social media (for further discussion, see [43]).

*MoodPrism* also collected data on the psychological characteristics of participants, which included personality, self-esteem, and social desirability. Multimedia Appendix 2 presents additional analyses, complementary to the findings presented here, exploring Facebook and Twitter user differences across these characteristics that may inform the patterns of emotion expressed over time.

### Data Analysis

**Figure 1:**
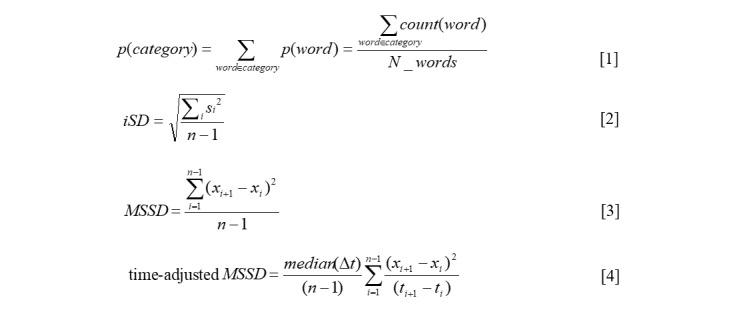
Equations for average proportion, within-person variability, and instability.

Within person variability, instability, and the average proportion of positive and negative emotion words in status updates on Facebook and Twitter were calculated for each participant. All equations are presented in Figure 1 and are defined below. Correlations with PHQ-9 scores were calculated. The distributions of all Facebook and Twitter variables were non-normal; consequently Spearman rho was selected for computing correlations. Exploratory post hoc comparison between the Twitter and Facebook samples on their psychological characteristics were also performed using Mann-Whitney *U* tests because of non-normal distributions. All analyses were performed in SPSS statistics, version 24 (IBM Corp) [53].

#### Average Proportion

Equation 1 in Figure 1 shows the relative proportion of positive and negative emotion words, which was calculated for each status update collected to adjust for the total number of words expressed, as described in Kern et al [43]. An average of these proportions was taken for each participant, resulting in the *average proportion of positive emotion words* and *average proportion of negative emotion words* across all status updates (range: 0-1), where *count(word)* refers to the total number of positive emotion words (or negative emotion words; the *LIWC 2007* category) contained in a status update, and *N_words* is the total number of words in that status update.

#### Variability

The within person variability (*iSD*) was computed for each participant across their status updates as shown in equation 2 in Figure 1, where the sum is taken over posts, *i*, *s*_i_ indicates deviations from the mean in an individual’s proportion of positive (or negative) words used in status updates, and *n* refers to the number of status updates for that individual. This resulted in the *positive emotion word variability* and *negative emotion word variability* across status updates for each participant.

#### Instability

The MSSD is defined for an individual in equation 3 in Figure 1, where *x*_i_ indicates the observation at index *i*, *x*_i+1_ refers to the next consecutive observation, and *n* refers to the total number of observations for that individual.

A major challenge in applying measures of time-structured variability to social media data is managing the irregularly spaced time intervals between posts. As observations on Facebook and Twitter occur in a natural setting, they often occur at irregular intervals spanning, for example, between hours and months. Thus, in addition to considering time order, the time elapsed between successive observations also needs to be considered. Emotion instability, when operationalized as MSSD, assumes even sampling of observations to be computed meaningfully [30,31]. Where this is not possible, adjustments can be applied to the data to provide a weighted estimate of time-structured variability [31]. As in the study by Jahng et al [31], a time-adjusted MSSD, which accounts for an uneven sampling of observations through time, was applied. This is shown in equation 4 in Figure 1, where *median(Δt)* is the median of incremental time differences across the whole recording period.

**Figure 2:**
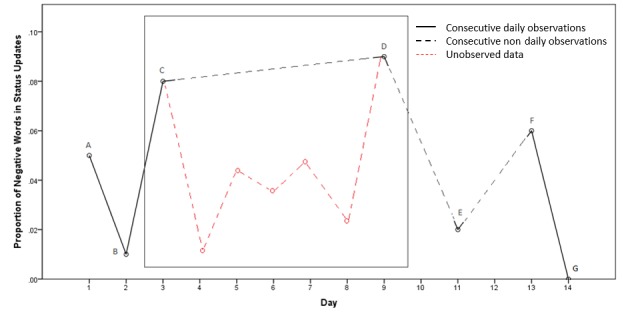
A simulated time series showing the proportion of negative emotion words used in status updates over 14 days. This irregularity of status updates (ie, missing observations on days 4-8 above) can be accounted for by reweighting pairs of observations by the time elapsed between them, resulting in a lower weight for the pair of points (points C and D). The observations within the box show similar levels of negative emotion word expression but occur 6 days apart and may appear to be temporally correlated if their relative temporal distance is not accounted for. The red points show the hypothetical unobserved fluctuations in negative affect that may have occurred during the intermediate 6 days.

This effectively makes observations closer together in time *more important* and those further apart *less important* to the reweighted MSSD statistic, relative to a participant’s median time increment between posts. Importantly, the time-adjusted MSSD, equation 4, reduces to the standard MSSD, equation 3, in the case that samples are spaced equally through time. The benefit of this adjustment in relation to social media data is the ability to utilize every observation without imposing strict inclusion criteria on the data (eg, a status update each day). As illustrated in Figure 2, this means that the overall variability contributed by all points can be included, and the potential contribution from points that may appear temporally correlated, if assumed to have occurred near in time (points C and D), is reduced when observations are in fact distant.

Applied to the Facebook and Twitter data for each participant, incremental time differences (*t*_i+1_*– t*_i_*)* between each status update were computed. The median of these time differences was then taken for each participant and applied to each incremental time difference and successive difference (*x*_i+1_*– x*_i_) in the proportion of positive or negative emotion words in a status update, as shown in equation 4. The average of the squared reweighted successive differences was then computed, resulting in the time-adjusted MSSD or the *positive emotion word instability* or *negative emotion word instability* across status updates. Here, greater values of time-adjusted MSSD indicate a greater magnitude of change in the proportion of emotion words expressed between all consecutive pairs of status updates relative to their median temporal separation.

## Results

### Sample Description

In total, 1856 status updates were collected (Facebook=538; Twitter=1,318) with 29,809 words expressed (Facebook=10,373; Twitter=19,436). In the Facebook sample, participants posted an average 18.55 (SD 10.01) status updates across the recording period; 55.8% (300/538) of the collected status updates contained positive emotion words, and 29.2% (157/538) contained negative emotion words. In the Twitter sample participants posted an average 26.90 (SD 11.71) status updates across the recording period; 63.6% (838/1318) of the collected tweets contained positive emotion words, and 56.2% (741/1318) contained negative emotion words.

Table 2 report the means, SDs, median, and interquartile range of the PHQ-9 scores and all Facebook and Twitter variables. It also presents descriptive statistics for the temporal aspects of posting status updates in the sample. Mann-Whitney *U* tests revealed no significant differences between Facebook and Twitter groups in the length of recording period sampled (*U*=590.50, *P*=.22), though there were differences in the median time difference (*U*=344.00, *P*<.001) and average number of status updates per day (*U*=509, *P*=.04), where Twitter users posted status updates more frequently and, based on their individual median, had smaller intervals in minutes between status updates.

Tables 3 and 4, respectively, report the two-tailed Spearman correlations (alpha level=.05) between the PHQ-9 and the positive emotion variables and the negative emotion variables from Facebook (above the diagonal) and Twitter (below the diagonal).

### Facebook Emotion Variability and Depression

Facebook users reported an average depression rating of 11.48 (SD 6.38) on the PHQ-9 and expressed 9.5% positive emotion words and 3.5% negative emotion words on average across their status updates. Depression severity was not significantly related to the average proportion of positive or negative emotion words expressed, positive or negative emotion word variability (*iSD*), or positive emotion word instability (time-adjusted MSSD). Negative emotion word instability did, however, show a significant positive association with depression severity ratings, sharing 19% of the variability. This indicates that successive status updates differed more in their proportion of negative emotion words used for individuals with higher self-reported depression symptoms.

**Table 2:** Descriptive statistics of the Patient Health Questionnaire-9 (PHQ-9), status update frequency, and the emotion features expressed in status updates on Facebook (n=29) and Twitter (n=49).

Variable		Facebook			Twitter		
		Range	Mean (SD)	Median (IQR^a^)	Range	Mean (SD*)*	Median (IQR)
Depression severity (PHQ-9^b^)		1-22	11.48 (6.38)	10 (5.5-17)	0-26	9.80 (6.81)	9 (4-14)
**Status update frequency**							
	Recording period (days)^c^	22-356	170.69 (116.05)	134 (54-290)	9-365	145.61 (124.97)	74.00 (33.50-272.00)
	Status updates per day	0.03-1.72	0.03 (0.36)	0.16 (0.07-0.51)	0.03-4.56	0.79 (1.09)	0.40 (0.09-0.90)
	Interval difference (min) between status updates^d^	661-34827	8446.65 (8724.25)	3818.00 (1877.75-13522.75)	4.0-28428.5	3939.79 (6616.84)	1037 (206.25-4571.25)
**Positive emotion words**							
	Average proportion	0.02-0.57	0.10 (0.10)	0.08 (0.05-0.11)	0.01-0.14	0.07 (0.03)	0.08 (0.05-0.09)
	Variability (*iSD*)^e^	0.04-0.47	0.13 (0.09)	0.10 (0.07-0.16)	0.03-0.17	0.07 (0.03)	0.08 (0.05-0.09)
	Instability (time-adjusted MSSD)^f^	0.003-11.54	1.14 (2.94)	0.11 (0.02-0.47)	0.0002-26.80	1.49 (4.40)	0.12 (0.02-0.83)
**Negative emotion words**							
	Average proportion	0.00-0.17	0.04 (0.04)	0.02 (0.01-0.05)	0.01-0.26	0.09 (0.06)	0.09 (0.04-0.12)
	Variability (*iSD*^e^)	0.00-0.31	0.07 (0.08)	0.03 (0.02-0.09)	0.02-0.14	0.08 (0.03)	0.08 (0.06-0.11)
	Instability (time-adjusted MSSD^f^)	0.00-1.23	0.11 (0.24)	0.01 (0.002-0.14)	0.0006-37.99	1.31 (5.43)	0.15 (0.03-0.49)

^a^IQR: interquartile range.

^b^PHQ-9: Patient Health Questionnaire-9.

^c^Recording period refers to the range of days between the first status update collected and the administration of the PHQ-9.

^d^The median interval differences between status updates.

^e^*iSD* refers to within-person variability.

^f^MSSD: mean squared successive difference.

**Table 3:** Spearman rho correlation analyses between depression severity (as rated by the Patient Health Questionnaire-9, PHQ-9) and the positive emotion features expressed in status updates on Facebook (n=29) and Twitter (n=49). Twitter correlations are shown *below* the diagonal; Facebook correlations are shown *above* the diagonal. CIs are reported at 95% and shown in brackets.

Variable		1	2	3	4
1. PHQ-9^a^		−	.04 (−0.33 to 0.40)	.17 (−0.21 to 0.51)	−.04 −0.40 to 0.33
2. Average proportion		.02 (−0.26 to 0.30)	−	.79^b^ (0.60 to 0.90)	.48^c^ (0.14 to 0.72)
3. Variability (*iSD*^d^)		−.09 (−0.36 to 0.20)	.49^b^ (0.24 to 0.68)	−	.61^b^ (0.31 to 0.80)
4. Instability (time-adjusted MSSD^e^)		−.20 (−0.46 to 0.09)	.31^c^ (0.03 to 0.54)	.48^b^ (0.23 to 0.67)	−

^a^PHQ-9: Patient Health Questionnaire-9.

^b^*P*<.001.

^c^*P*<.05.

^d^*iSD* refers to within-person variability.

^e^MSSD: mean squared successive difference.

**Table 4:** Spearman rho correlation analyses between depression severity (as rated by the Patient Health Questionnaire-9, PHQ-9) and the negative emotion features expressed in status updates on Facebook (n=29) and Twitter (n=49). Twitter correlations are shown below the diagonal; Facebook correlations are shown above the diagonal. CIs are reported at 95% and shown in brackets.

Variable		1	2	3	4
1. PHQ-9^a^		−	.12 (−0.26 to 0.46)	.20 (−0.18 to 0.53)	.44^b^ (0.09 to 0.69)
2. Average proportion		−.14 (−0.41 to 0.15)	−	.95^c^ (0.90 to 0.98)	.72^b^ (0.48 to 0.86)
3. Variability (*iSD*^d^)		−.36^b^ (−0.58 to 0.09)	.57^c^ (0.34 to 0.73)	−	.82^c^ (0.65 to 0.91)
4. Instability (time-adjusted MSSD^e^)		−.20 (−0.46 to 0.09)	.28 (−0.001 to 0.52)	.49^c^ (0.24 to 0.68)	−

^a^PHQ-9: Patient Health Questionnaire-9.

^b^*P*<.05.

^c^*P*<.001.

^d^*iSD* refers to within-person variability.

^e^MSSD: mean squared successive difference.

**Figure 3:**
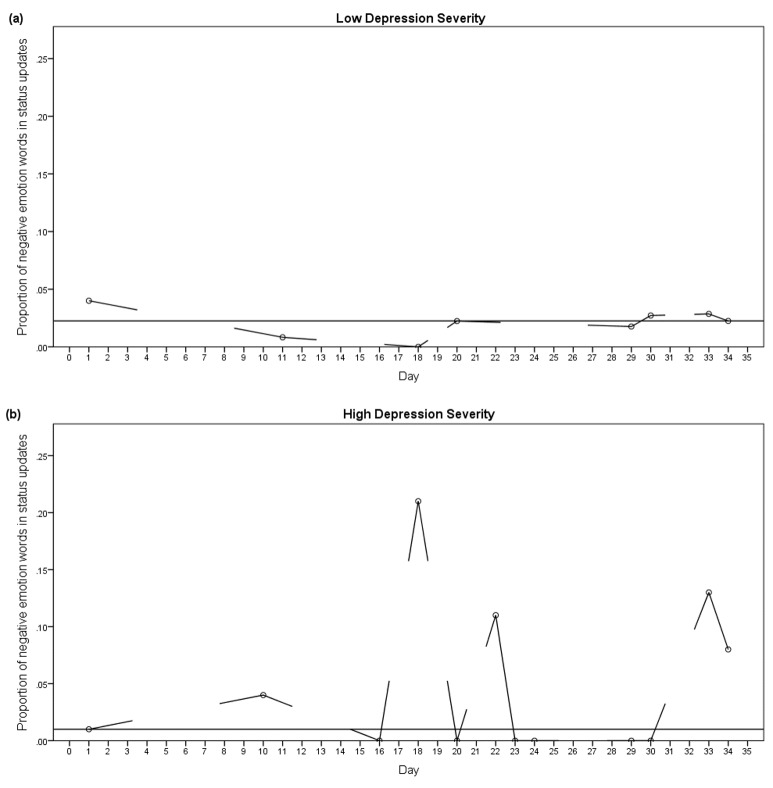
Graphs showing the proportion of negative emotion words used in individual status updates on Facebook across 35 days. (a) Shows an individual with low self-reported depression severity (Patient Health Questionnaire-9, PHQ-9 score=9) who demonstrated little post-to-post variation in the proportion of negative emotion words used, with the maximum difference of .03. The horizontal trend line shows the median proportion of negative emotion words used (.022) and interpolation lines link consecutive status updates. (b) Shows an individual with high self-reported depression severity (PHQ-9 score=22), who demonstrates large post-to-post changes in the proportion of negative emotion words used in status updates with the largest difference being .21. The horizontal trend line shows the median proportion of negative emotion words used (.01) and interpolation lines link consecutive status updates.

When controlling for the average proportion of negative emotion words expressed in status updates, negative emotion word instability remained strongly associated with depression severity (partial Spearman correlation: *r*_s_(26)*=*.51, *P*=.006). Similarly, when controlling for negative emotion word variability, negative emotion word instability remained strongly associated with depression severity (*r*_s_(26)=.49, *P*=.009). To illustrate this effect, Figure 3 shows samples of the pattern of negative emotion word instability from 2 participants; one with low and one with high self-reported depression symptoms. As can be seen in Figure 3, the individual with low depression severity shows small magnitude changes in their use of negative emotion words across status updates. In contrast, the individual with high depression severity exhibits greater magnitude spikes in negative emotion word expression. Here, instability is independent of variability (see Multimedia Appendix 3 for consideration of instability under fixed variability conditions).

### Twitter Emotion Variability and Depression

Twitter users reported an average depression rating of 9.80 (SD 6.81) on the PHQ-9 and expressed 7.4% positive emotion words and 9.2% negative emotion words on average across their status updates. Depression severity was not significantly related to the average proportion of positive or negative emotion words expressed, positive emotion word variability (*iSD*), or positive or negative emotion word instability (time-adjusted MSSD). Negative emotion word variability, however, was significantly negatively associated with depression severity ratings, sharing 13% of the variability. That is, a greater general dispersion of negative emotion across status updates on Twitter was associated with lower depression severity. When controlling for the average proportion of negative emotion words expressed in status updates, negative emotion word variability retained its association with depression severity in a partial Spearman correlation *r*_s_(46)=−.35, *P*=.01.

**Figure 4:**
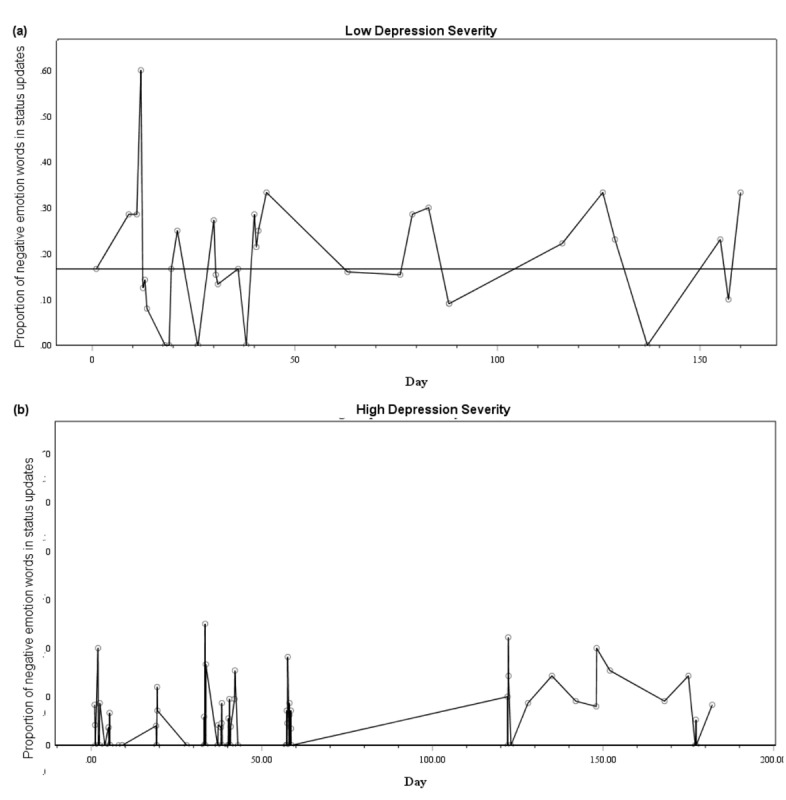
Graphs showing the proportion of negative emotion words used in individual status updates across (a) 160 and (b) 182 days. (a) Shows an individual with low self-reported depression severity (Patient Health Questionnaire-9, PHQ-9 score=8) and high variability in the proportion of negative emotion words used across their recording period. The horizontal trend line shows the median proportion of negative emotion words (.17) and interpolation line links status updates. (b) Shows an individual with high self-reported depression severity (PHQ-9 score=16) and low variability in the proportion of negative emotion words used across their recording period. The median proportion of negative words used was .00 and is therefore not shown. The interpolation line links status updates.

To illustrate this effect, Figure 4 shows samples of the pattern of negative emotion word variability from 2 participants; one with low and one with high self-reported depression symptoms. As can be seen in Figure 4, the individual with low depression severity shows larger overall variability in their use of negative emotion words across status updates. In contrast, the individual with high depression severity exhibits more restricted variability in negative emotion word expression. It is important to note that in Figure 4, tweets often occurred on the same day, which accounts for the clustering in the figure.

### Facebook and Twitter Status Update Frequency and Depression

Descriptive statistics for the average number of status updates per day and the median time interval between status updates are presented in Table 2. Spearman correlations revealed a significant positive association between the average number of status updates per day and depression severity for Facebook users, *r*_s_(29)*=*.48, *P*=.008. There was also a significant negative association between the median time interval between status updates and depression severity for Facebook users, *r*_s_(29)*=* −.61, *P*<.001. Depression severity was not significantly related to the average number of status updates per day or the median interval between status updates for Twitter users.

### Differences in Emotion Language Patterns

As indicated in Tables 3 and 4, the pattern of relationships between depression and emotion language use varied between Facebook and Twitter users. To explore this further, comparisons of the social media emotion language variables between the samples were conducted. As all variables were nonnormally distributed, Mann-Whitney *U* tests were used to compare the mean rank differences between Facebook and Twitter users in their emotion language patterns. The Twitter sample expressed more negative language (*U*=256.00, *P*<.001) that was more variable (*U*=400.00, *P*=.001) and unstable *(U*=379.00, *P*=.001) than did the Facebook group across the recording period. Twitter users also expressed greater variability in their positive emotion compared with Facebook users (*U*=413.00, *P*=.002).

## Discussion

### Principal Findings

This study aimed to determine whether emotion variability and instability across status updates on Facebook and Twitter are useful indicators of depression. Differences between the social media platforms were also explored. The findings suggest that instability in the negative emotion content across Facebook status updates may indeed be a useful indicator for depression and that the time-adjusted MSSD is an effective index of instability that accounts for the uneven temporal sampling of social media posts. As hypothesized, negative emotion word instability retained its association with depression severity when the average proportion of negative emotion word use and negative emotion word variability were controlled. This index may provide additional sensitivity over basic frequency indices that are typically used in social media and depression studies. However, negative emotion word instability did not emerge as a predictor of depression on Twitter. Rather, in contrast to expectations, negative emotion word variability was negatively associated with depression severity. Furthermore, the average proportions of negative and positive emotion word use were not significantly associated with depression severity on either Facebook or Twitter. Other temporal features, the average number of status updates per day, and the median time interval between status updates were also associated with depression severity, but only for Facebook users.

### Negative Affect Instability on Facebook

Greater negative emotion word instability on Facebook was associated with individuals experiencing greater depression severity. The time-adjusted MSSD scores were driven by the pattern of frequent, high magnitude changes in negative emotion word use between status updates, not variability alone. This finding is consistent with previous studies measuring negative affect through self-report over time that have demonstrated negative affect instability to be predictive of depression [25,31,33-36].

Many users post on Facebook to broadcast emotion [14], and emotion words are often present in posts [15]. Individuals with depression are more likely to produce more content on social media when experiencing more severe symptoms [16,17], and this often relates to the disclosure of symptoms, negative experiences, or posting to seek social support [3,8,10,11]. Indeed, this was reflected in the current sample where Facebook users with greater depression severity ratings posted more status updates per day, more frequently (ie, there was a smaller median time interval between consecutive status updates). Large changes in the proportion of negative emotion words used between consecutive status updates could reflect patterns of Facebook use that mirror the inherent variation in the severity of depression symptoms over time. In this way, the negative emotion word instability in the status updates on Facebook may reveal the ebb and flow of depression symptoms and emotion dysregulation in daily life [36].

Negative emotion word instability on Facebook may also be tied to specific events, capturing momentary responses to internal and external stressors. Individuals exposed to an emotional event generally post status updates in a mood congruent way (eg, happy or sad) [54]. The proportion of negative emotion words used in a status update may reflect the extremity with which an event is perceived as negative or positive. In this light, status updates could provide insight into emotional reactivity to events. A depression-specific pattern of instability in status update expression on Facebook may exist that reflects the amplification of negative emotion in response to ambiguous or negative events [55] and a mood brightening effect in response to positive stimuli, where there is a large reduction in expressed negative affect [36,56].

The unique fluctuating pattern of negative emotion expression in individuals with more severe depression symptoms was further supported by negative emotion word instability, which remained associated with greater depression symptoms when controlling for the average proportion of negative emotion words used in status updates. This suggests that the time-structured patterns of emotion expressed on Facebook may provide better differentiation between individuals with and without depression where they express similar levels of negative emotion words. This study suggests that the poor hit rate in some keyword approaches to classifying depression in status updates, as described by Mowery et al [18], may be enhanced by including measures of moment-to-moment variability in emotion word use.

### Within-Person Variability in Emotional Expression on Twitter

Contrary to expectations, Twitter users who had lower variability in their use of negative emotion words across the recording period were more likely to have greater self-reported depression severity. This sits in contrast with a recent meta-analysis that showed negative emotion variability shares a positive association with depression [1].

It could be that the greater variability in emotion expressed by individuals lower in depression on Twitter reflects adaptive emotional functioning. In addition to personal disclosures and using Twitter to talk about daily events [57], people turn to Twitter to post content about politics, world events, and to share information [58]. Expressing a wide range of negative emotion in response to these diverse personal and community-related events may be appropriate to the context or be a part of effective emotion regulation strategies. Indeed, expressive emotional writing has been linked to better psychological and physical outcomes in offline and online settings [59-62].

On the other hand, Twitter users with higher levels of depression expressed a more clustered spread of negative emotion. Emotion appraisals of internal and external events and their subsequent expression in status updates may be more restricted or blunted for Twitter users with higher levels of depression. This is consistent with studies indicating that MDD is associated with reduced emotion reactivity [63].

### Variability Differences Between Facebook and Twitter

Two divergent emotion patterns relating to depression emerged from the Facebook and Twitter samples. This highlights the importance of collecting data from multiple social media platforms, as differences in the communication mechanisms and population demographics across social media sites greatly impacts on the generalizability of findings [49]. In terms of emotion expression, Twitter users expressed more negative emotion that was more variable across the recording period than Facebook users. This may be because of the 140-character restriction placed on tweets (recently increased to 280 characters) [64] compared with the 63,206-character limit on Facebook [65], which may impact on the total proportion of emotion words expressed and the magnitude of change observed between posts. On Twitter, when an emotion word is used, it is likely to occur in the context of fewer total words and will result in a greater proportion emotion expressed per Tweet. In contrast, when a Facebook user expresses emotion, it may occur in the context of more total words, potentially reducing the overall proportion of emotion words expressed.

Other confounding variables may also create differences between negative emotion expression on Facebook and Twitter. For example, Twitter allows users to generate anonymous accounts, whereas Facebook accounts are likely to be linked to a real name. The anonymity may release the user from social norms and increase expression of negative emotion [66,67]. Twitter also is less symmetrical, with weaker relational ties, and less dense network structures, which impacts on the emotion people express to their networks [68,69]. These different social contexts and related norms are a fruitful area for future research.

### Averages of Negative and Positive Emotion Word Use Are Not Associated With Depression

Inconsistent with many previous findings [2,3,5,9], the average proportion of positive and negative emotion words used across status updates on both Facebook and Twitter were not significantly associated with depression. Other approaches using the *LIWC 2007* positive and negative dictionaries have found that as negative emotion expression increases, so does the ratings of self-reported depression severity (eg, [5]). This could be due in part to the small sample used here; language is noisy [43], and with only 29 and 49 participants in the Facebook and Twitter samples, respectively, the signal may not be enough to counteract that noise (see Kern et al [43] for further consideration of language and sample size considerations). Among this noise, it is notable with the small number of participants that a robust association between negative affect instability and depression on Facebook was found, suggesting a strong relationship between these two quantities. Although this result needs to be replicated in other samples, this suggests that when a smaller number of participants are available, instability may be a more sensitive measure than frequency in detecting depression severity.

The null findings between depression and the average proportion of words in status updates may also reflect the lack of precision that frequency measures provide. As shown by Mowery et al [18], using a keyword approach to identifying depression in social media posts results in a large proportion of false-positives, reducing the specificity with which depression can be identified through the average emotion expressed over time. Context matters [43], such that the use of a word may not directly link to an experienced emotion (eg, “I went to visit Happy Valley” does not indicate positive emotion). It is important to acknowledge also that negative emotion expression is not the exclusive domain of individuals with higher levels of depressive symptoms. It is also possible that in this study, the amended negative emotion word dictionary of the *LIWC 2007* alone was not sufficient in identifying the words most indicative of depression. Indeed, the dictionaries were recently updated [70], and future studies should examine whether the updated *LIWC 2015* dictionaries offer a better indication of depression. Personality, gender, and age have all previously been shown to impact on the number of negative emotion words people use online (cf [71]), and this complexity might also be considered in future research.

### Limitations and Future Directions

There are several limitations to this study. First, although emotion scores were calculated, the actual posts were not available (because of privacy considerations), such that the context of their content could not be considered. It is therefore possible that some posts may have obtained a negative emotion word count where a positive message was conveyed. Future research should seek to apply more sophisticated open vocabulary approaches or postprocessing of status updates [18,43] to provide greater detail and accuracy of the language use context.

Second, the sample analyzed was small, and this may have impacted on the power to detect significant associations between variables. This may have obscured potential associations between the expression of negative emotion words and depression severity. It is also likely that, because of sample size, the findings obtained here may not be generalizable to the Facebook and Twitter populations. Replication is required in larger samples.

Third, only original posts were used, with retweets or shared posts excluded. Although retweets may be an indirect indication of a person’s emotions, beliefs, values, and behaviors, the intentions underlying reposting are unclear. Furthermore, at the time of data collection, reposting updates was less common in Facebook, so excluding retweets provided a clearer comparison. Future studies might explore the extent to which reposts (retweets and the sharing of posts) reflect a user’s values and emotions and indicate depression status.

It is also important to note that it was unknown if the emotion words expressed on Facebook or Twitter accurately reflected same-day subjective changes in mood. Further research should seek to link consecutively measured mood ratings with social media data to strengthen the assumption that interpretation of social media content reflects real-world emotion experience.

Finally, studies should seek to explicitly consider inertia in the emotion expressed in status updates as a predictor of depression and consider how the sensitivity and accuracy of frequency and instability metrics changes across different sample sizes. Such analyses will, however, require adjustments be made to calculations to account the sparseness and irregularity of social media data.

### Conclusions

This study suggests that instability in the negative emotion expressed on Facebook provides insight into the presence of depression symptoms for social media users, and greater variability of negative emotion expression on Twitter may be protective for mental health.. If replicated in other samples, emotion dynamics might be applied to big data approaches for depression screening at a population level, providing insight into the emotion processes underlying depression and improving the specificity of depression identification above using language averages alone. The time-adjusted MSSD appropriately accounts for the uneven temporal sampling of real-world social media data, providing a sensitive measure of emotion instability that may be used as an early indicator of (or identified as a risk factor for) depression. Variability is often seen as a nuisance factor that creates noise and obscures other associations. Treating emotion variability as a legitimate individual difference may be an important step in better describing the microprocesses that lead to psychopathology. The findings also point to possible differences across the online culture created by a particular social media platform, such that different platforms may provide different insights into mental health.

The widespread and frequent use of social media has generated considerable concern around its impact on mental health. Yet, social media is also revealing itself to be a valuable avenue for the ongoing monitoring of depression. This study contributes to understanding the best approaches for using the technology to help users suffering from depression.

## Appendix

Multimedia Appendix 1 Emoji and internet slang supplements to the LIWC 2007 dictionaries.

Multimedia Appendix 2 Supplementary analyses: personality, self-esteem, and social desirability.

Multimedia Appendix 3 Illustrative participant data showing instability at fixed levels of variability.

## Abbreviations

ESM: experience sampling method
LIWC 2007: Linguistic Inquiry and Word Count 2007
MDD: major depressive disorder
MSSD: mean squared successive difference
PHQ-9: Patient Health Questionnaire-9
