# Supplementary material for: Predicting Depression From Language-Based Emotion Dynamics: Longitudinal Analysis of Facebook and Twitter Status Updates
Source: J Med Internet Res. 2018 May 8;20(5):e168. doi: 10.2196/jmir.9267 (PMC5964306; doi:10.2196/jmir.9267)
Supplement: Multimedia Appendix 1 [file jmir_v20i5e168_app1.pdf]

Multimedia Appendix 1

Emoji & internet slang additions to the *LIWC 2007* emotion dictionaries

Positive Emoji’s	Negative Emoji’s		Positive Emotion Internet Slang			Negative Emotion Internet Slang	
=D>	:-l	:-/	Aight	j/j	lyke	Abft	fomo
=D	:l	:/	Aightz	jk	marvy	Acgaf	gank
:D	X(	T_T	Aiight	j/k	njoy	Aiic	grmbl
:-D	X-(	T^T	Aite	jks	ql	Arsed	h8
(:)	O	QQ	Alrt	j2f	qool	Awk	h80r
(:-)	b (	=O::::	alryt	j4f	qt	Bm	h83r
>:D<	>:-(	=’(	Alol	j4g	qte	Boom	h8ed
:))	>:-)	&.(..	Aml	j4l	rofl*	Bord	h8r
:-X	(:-&	(;_;)	Aprece8	jfg	*s*	Bovered	h8red
:X	:@	:’(	Apreci8	jfl	S’ok	Catwot	h8s h8t
:)	:(	:’	Awes	jfk	Snog	Cba	h8t0r
:-)	:-t	:’-(	Awsm	jja	Sok	Cbb	h8t3r
^^	:-ll	:*(	Awsome	jkz	Sof	Cbf	h8te
:,’)	:ll	:*-(	Bahaha	k	Stm	Cbfa	h8tr
:-“)	:t	:_(	Besos	kay	Ub3r	Cbfed	hait
:”)	:-@	:..[	Bilu	kewel	W00t	Cmeo	heyt
;)	:{	;_;	Btwilu	kewl	Woot	Cmw	htr
;-)	:-Z	=\	Btwitiailwu	kk	Xellent	Cof	id10d
\o/	:Z	:l	Byak	kl	Xlnt	Cotf	idjit
;-(	??	:(&	Chillin	koo	xtc	Dc	idot
;(	@_@	:-E	Ctm	kool		Dbm	idyat
:’)	(-_-*)	:-e	Cut3	kss		Dfc	invu
:’-)	(o_o)	DX	Eil	kssd		Dgac	irhy
>-)	b-(	/:(	Fab	kul		Dgaf	isb
:p	%+l	:-[	Fah	kute		Dgara	kmn
:P	:S	:[	Fi9	l0lz		Dgas	kmp
:-P	:-S	:-}	Funee	laff		Eejit	lsr
:-p	(:-	:}	Funy	lal		Eedyat	lzr
:>	:(	#-)	*g*	lol		Ef	nv
:->	:-(	#)	Gtm	lols		Ef-ing	ofcol
l__l	l-o	:-6	Gud	lel		Effed	oh noes
^.^	\-o	:6	Gudd	liek*		Effin	oh noez
^?^	:-o	:-@	Heh	lik		Effing	p/oed
^-^	=((	(:(	Hppy	lk		F#cking	poed
%)	+-((	:-<	Hpy	lke		F’n	ph33r
%-)	</3	><	Haha	llc		f-ing	phayl
<3	(U)	://	I8	llf		fml	phail
<u3	:-c		Iatb	lmao		f.m.l	r8p
	:c		Iight	lmfao		farg	r8pist
	O.o		Iigh	rofl		fck	stupd
	%)		Ijdl	lml		fcked	suk
	%-)		Ili	lov		fckin	sukz
	(@_@)		Ilml	luv		fcking	suxx
	:s		Ilms	luff		feck	wrdo
	:-Q		Ilu*	luvv		fk	wtf
	:Q		Ilshipmp	lurve		fkd	
	:-$		Irly	lv		fker	
	:$		Ite	lve		fkin	
			Jj	lyk		fking	
