# Supplementary material for: Predicting Depression From Language-Based Emotion Dynamics: Longitudinal Analysis of Facebook and Twitter Status Updates
Source: J Med Internet Res. 2018 May 8;20(5):e168. doi: 10.2196/jmir.9267 (PMC5964306; doi:10.2196/jmir.9267)
Supplement: Multimedia Appendix 2 [file jmir_v20i5e168_app2.pdf]

Multimedia Appendix 2

Supplementary Analyses: Personality, Self-esteem, and Social Desirability

Supplementary analyses were conducted to explore the individual characteristics that may be involved in the different emotion expression patterns between Facebook and Twitter.

Measures

MoodPrism collected data on the psychological characteristics of participants which included personality, self-esteem, and social desirability.

Personality, based on the Five Factor Model (extraversion, neuroticism, conscientiousness, agreeableness, and openness to experience), was measured by the Mini-IPIP scales [1]. The Mini-IPIP has 20-items, with four items addressing each personality factor. Example items include: Extraversion (e.g. "I am the life of the party"); Neuroticism (e.g. "I have frequent mood swings"); Conscientiousness (e.g. "I get chores done straight away"); Agreeableness (e.g. "I sympathize with others' feelings"); and Openness (or Intellect/Imagination) (e.g. "I have a vivid imagination"). Statements are rated on a 5-point Likert scale ranging from "1- Very Inaccurate" to "5 - Very Accurate", with a mid-point of "3 – Neither agree nor disagree). The five-factor structure of the Mini-IPIP has been supported across several studies and has adequate internal reliability for all sub-scales (α's > .60) [1,2].

Self-esteem was measured with the Rosenberg Self-Esteem Scale (RSES) [3]. Participants rate 10 statements about their feelings toward themselves on a 4-point Likert scale from "0 – Strongly Disagree" to "3 – Strongly Agree". Five items relate to positive feelings (e.g. "I feel that I have a number of good qualities"), and five are negative and are reverse scored (e.g. "I certainly feel useless at times"). Ratings are summed to generate a total score ranging between 0-30 where higher scores indicate greater self-esteem. The RSES demonstrates good reliability across a range of demographic characteristics (α = .91) [4].

Social desirability, the tendency with which an individual presents a desirable impression of themselves, was measured by the short form Marlowe-Crowne Social Desirability Scale Form C (M- C Form C) [5]. Participants respond to 13 dichotomous items describing socially desirable (but rare) or undesirable (but common) behaviours (e.g. "No matter who I'm talking to, I'm always a good listener"). These are rated as being "True" or "False" (items are reverse-scored to the socially-desirable direction) and are summed to create a score ranging from 0-13, where higher scores indicate a greater tendency to present a socially desirable image of oneself to others. The M-C Form C has demonstrated good reliability (Kuder-Richardson r = .76) [5].

Results

Table 1 presents the means and standard deviations of the psychological characteristics for participants with available data in the Facebook and Twitter samples.

Table 1

Means and standard deviations of psychological characteristics in the Facebook and Twitter samples

**Variable** [a]	**Scale Range**	**Platform**	
		Facebook *M (SD)*	Twitter *M (SD)*
Depression Severity	0 – 27	11.48 (6.38)	9.80 (6.81)
Extraversion	1 – 20	8.95 (3.02)	10.34 (4.50)
Agreeableness	1 – 20	14.35 (3.69)	15.34 (4.09)
Conscientiousness	1 – 20	11.48 (3.57)	13.59 (3.69)
Neuroticism	1 – 20	14.39 (3.27)	13.02 (4.61)
Openness to Experience	1 – 20	12.87 (2.67)	13.10 (3.27)
Self-Esteem	0 - 30	14.05 (2.09)	15.30 (1.91)
Social Desirability	0 - 13	5.13 (2.69)	6.49 (2.50)

[a] Sample sizes differ across variables. For Facebook *n* ranges between 21 -29; for Twitter *n* ranges between 40 – 49.

As all distributions were non-normal, Mann-Whitney U tests were selected to compare mean ranks on psychological characteristics between the Facebook and Twitter users. No significant differences were observed in depression severity, extraversion, agreeableness, neuroticism, or openness to experience. Twitter users did have significantly higher mean ranks in conscientiousness (*U* = 288.50, P = .03), self-esteem (*U* = 250.00, P = .02), and social desirability (*U* = 419.00, P = .046) compared to Facebook users.

Discussion

Compared to the Twitter sample, Facebook users had lower, conscientiousness, self-esteem, and lower scores of social desirability. Lower levels of conscientiousness have associated with less impulse control and can result in greater reactivity in negative emotion [6]. Combined with a reduced tendency to present a positive self-image to others, this may account for the negative emotion instability observed on Facebook but not Twitter; particularly as low conscientiousness also plays as role in less cautious online behaviour [7, 8]. This may elicit status updates on Facebook directly tied to emotional experiences with less reflective construction. Twitter users in contrast may be more inclined to monitor their online emotion expression and present more favourable representations of themselves. Though we

lacked the power to control for these characteristics in regression analyses they may be informative targets for future research.

References

1. Donnellan MB, Oswald FL, Baird BM, Lucas RE. The Mini-IPIP Scales: Tiny-yet-effective measures of the Big Five Factors of Personality. *Psychol Assess*. 2006;18(2):192-203. doi:10.1037/1040-3590.18.2.192.

2. Baldasaro RE, Shanahan MJ, Bauer DJ. Psychometric Properties of the Mini-IPIP in a large, nationally representative sample of young adults. *J Pers Assess*. 2013;95(1):74-84. doi:10.1080/00223891.2012.700466.

3. Rosenberg M. *Society and the Adolescent Self-Image*. Princeton, NJ: Princeton University Press; 1965. doi: 10.1126/science.148.3671.804

4. Sinclair SJ, Blais MA, Gansler DA, Sandberg E, Bistis K, LoCicero A. Psychometric properties of the Rosenberg Self-Esteem Scale: Overall and across demographic groups living within the united states. *Eval Health Prof*. 2010;33(1):56-80. PMID: 20164106.

5. Reynolds WM. Development of reliable and valid short forms of the Marlowe-Crowne social desirability scale. *J Clin Psychol*. 1982;38(1):119-125. doi:10.1002/1097-4679(198201)38:1<119::AID-JCLP2270380118>3.0.CO;2-I.

6. Fayard JV, Roberts BW, Robins RW, Watson D. Uncovering the affective core of conscientiousness: The role of self-conscious emotions. *J Pers*. 2012;80(1):1-32. PMID: 21241309.

7. Hollenbaugh EE, Ferris AL. Facebook self-disclosure: Examining the role of traits, social cohesion, and motives. *Comput Hum Behav*. 2014;30:50-58. doi:10.1016/j.chb.2013.07.055.

8. Seidman G. Self-presentation and belonging on Facebook: How personality influences social media use and motivations. *Personal Individ Differ*. 2013;54(3):402-407. doi:10.1016/j.paid.2012.10.009.
