# Supplementary material for: Predicting Depression From Language-Based Emotion Dynamics: Longitudinal Analysis of Facebook and Twitter Status Updates
Source: J Med Internet Res. 2018 May 8;20(5):e168. doi: 10.2196/jmir.9267 (PMC5964306; doi:10.2196/jmir.9267)
Supplement: Multimedia Appendix 3 [file jmir_v20i5e168_app3.pdf]

Multimedia Appendix 3

Illustrative participant data showing instability at fixed levels of variability.

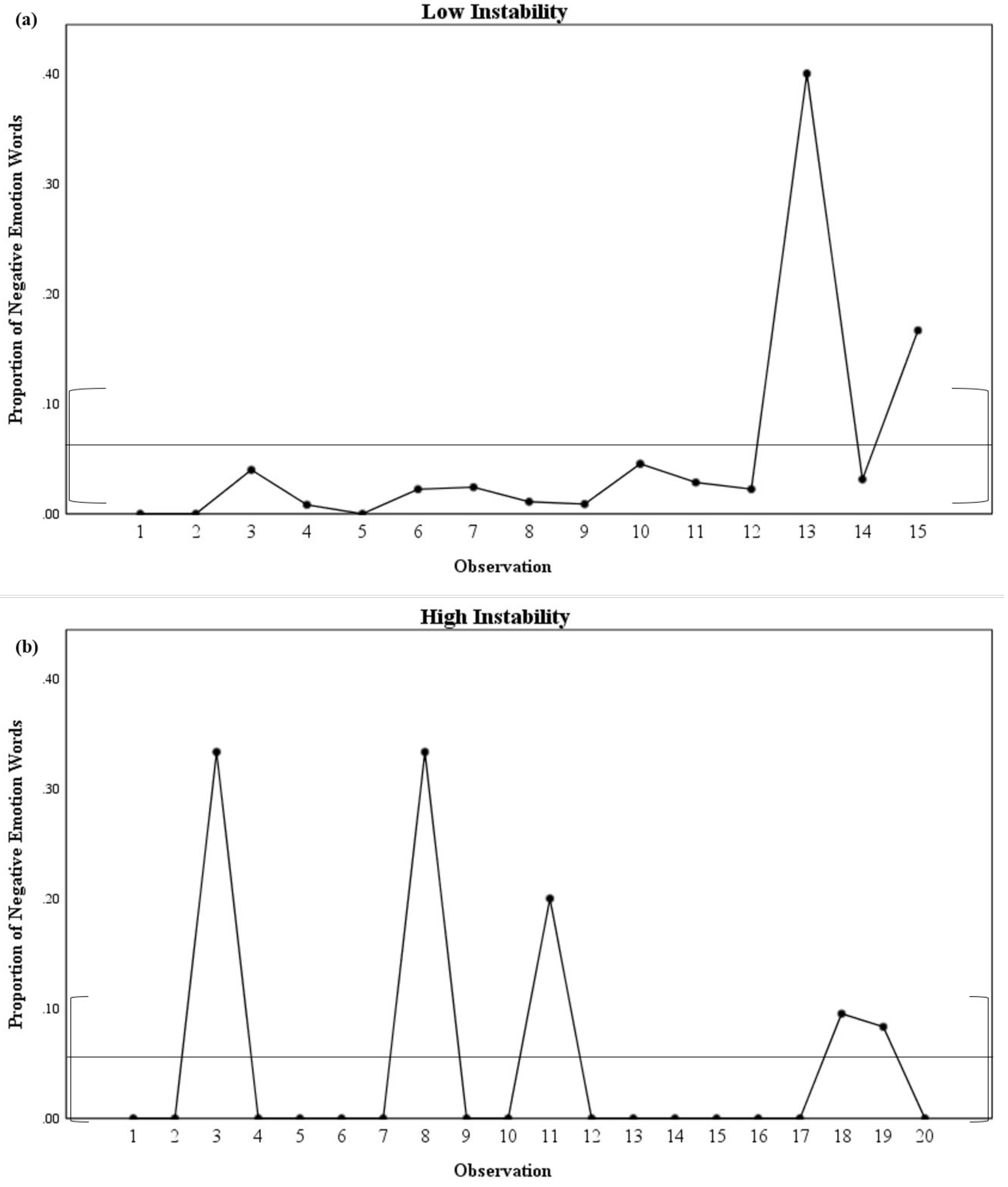

Figure 1. Example participant data showing instability under fixed variability (*iSD)* conditions. Panel (a) shows a participant with a low instability value (.015) and variability of .104. Panel (b) shows a participant with a similar variability value (.108) and a high instability value (.290). Brackets show the *iSD* around the mean (horizontal trend line). Time-scale is not shown here to compress Facebook record into a visible format for both participants.
